# First continuous pre-Jaramillo to Jaramillo terrestrial vertebrate succession from Europe

**DOI:** 10.1038/s41598-020-58404-w

**Published:** 2020-02-05

**Authors:** Pedro Piñero, Jordi Agustí, Oriol Oms, Hugues-Alexandre Blain, Marc Furió, César Laplana, Paloma Sevilla, Antonio Rosas, Josep Vallverdú

**Affiliations:** 1 IPHES, Institut Català de Paleoecologia Humana i Evolució Social, Zona Educacional 4, Campus Sescelades URV (Edifici W3), 43007 Tarragona, Spain; 2 Sección Mastozoología, Museo de La Plata (UNLP), CONICET, Paseo del Bosque s/n, 1900 La Plata, Buenos Aires, Argentina; 3 ICREA, Institució Catalana de Recerca i Estudis Avançats, Pg. Lluís Companys 23, 08010 Barcelona, Spain; 4 Àrea de Prehistòria, Universitat Rovira i Virgili (URV), Avinguda de Catalunya 35, 43002 Tarragona, Spain; 5 Departament de Geologia, Universitat Autònoma de Barcelona, 08193 Bellaterra, Spain; 6 Institut Català de Paleontologia Miquel Crusafont, Universitat Autònoma de Barcelona, Edifici ICTA-ICP, Carrer de les Columnes s/n, Campus de la UAB, 08193 Cerdanyola del Vallès, Barcelona Spain; 7 MAR, Museo Arqueológico Regional de la Comunidad de Madrid, Plaza de las Bernardas s/n, 28801 Alcalá de Henares, Spain; 8 Departamento de Geodinámica, Estratigrafía y Paleontología, Facultad de Ciencias Geológicas, Universidad Complutense de Madrid, Ciudad Universitaria, 28040 Madrid, Spain; 9 Departamento de Paleobiología, Museo Nacional de Ciencias Naturales (CSIC), José Gutiérrez Abascal 2, 28006 Madrid, Spain

**Keywords:** Palaeomagnetism, Palaeontology

## Abstract

In this paper, the early Pleistocene small vertebrate sequence of Quibas-Sima (Quibas karstic complex, Murcia, SE Spain) is presented. The available magnetostratigraphic information together with the small vertebrate association, allow to reliably constrain the age of the different units. The basal unit of the section has recorded a reversed polarity assigned to the pre-Jaramillo Matuyama (C1r.2r, i.e., between 1.2 and 1.07 Ma). The intermediate units have recorded a normal polarity correlated directly with the Jaramillo subchron (C1r.1n, between 1.07 and 0.99 Ma), while the upper units record the post-Jaramillo reverse polarity (C1r.1r, i.e., between 0.99 and 0.78). Jaramillo subchron is especially significant regarding the earliest hominin dispersal in Western Europe. However, vertebrate faunas unambiguously correlatable with Jaramillo subchron are extremely rare in Europe. Thereby, the study of the Quibas-Sima sequence allows to characterize the vertebrate association synchronous to this paleomagnetic episode in southern Iberian Peninsula, and contributes to increase knowledge of the biotic and climatic events that took place in southern Europe at the beginning of the Early-Middle Pleistocene transition, prior to the Matuyama-Brunhes boundary. A continuous small vertebrate succession has been reported, including representatives of the families Bufonidae, Pelodytidae, Testudinidae, Gekkonidae, Blanidae, Lacertidae, Colubridae, Viperidae, Soricidae, Erinaceidae, Rhinolophidae, Vespertilionidae, Muridae, Gliridae, Sciuridae, Leporidae and Ochotonidae The ecological affinities of the faunal association suggest a progressive reduction in forest cover in the onset of the Jaramillo subchron.

## Significance

The Jaramillo subchron (1.07–0.99 Ma) is a normal polarity episode reported within the upper part of Matuyama Chron. This paleomagnetic interval characterizes the early phase of the Early-Middle Pleistocene transition (∼1.2–0.5 Ma), a time span that represents an important change in the Earth’s climate. Moreover, this episode is particularly relevant concerning the earliest hominin occurrence in Western Europe. Unfortunately, European terrestrial vertebrate sequences recording the Jaramillo subchron are very rare. The combination of biochronology and magnetochronology has vantage to precisely constrain the geochronology of the studied site. On the basis of a sound taxonomy of the small vertebrate fauna and solid magnetostratigraphy, the Quibas-Sima section (Spain) is here dated between 1.2 and 0.78 Ma. This implies that, for the first time in Europe, a continuous continental succession recording pre-and syn-Jaramillo vertebrate faunas is reported. For this reason, Quibas-Sima offers an excellent opportunity to increase our knowledge about the faunal and paleoenvironmental context of the time span comprised between roughly 1.2 and 0.99 Ma (Epivillafranchian). This paper provides new data that will help to understand better faunal composition of ecosystems in southwestern Europe during a relevant time interval such as the late early Pleistocene and its paleoclimatic implications.

## IntroductionIntroduction

The subchron C1r-1n (1.07–0.99 Ma)^1^, better known as Jaramillo, is a very significant stratigraphic marker within the early Pleistocene. The Jaramillo subchron covers MIS 28–31. This interval is of great interest and relevance in relation to the first occurrence of hominin presence in Western Europe (Barranco León 5, Fuente Nueva 3, Sima del Elefante 9c, Le Vallonet)^2–5^. Unfortunately, early Pleistocene small vertebrate successions with a well dated framework immediately prior or younger than this paleomagnetic reversal are scarce in Europe^6–9^, whereas small vertebrate faunas unambiguously correlatable with Jaramillo subchron are extremely rare^10,11^. In Eastern Europe, small mammals from the Ostrogozh layer of the Korotoyak section (Russia), according to paleomagnetic evidence, were correlated with the Jaramillo subchron^12^. In Western Europe, the Colle Curti sequence (central Italy) covers a part of the Matuyama epoch which includes the Jaramillo subchron^13^. The Castagnone section (northern Italy) recorded normal polarity deposits with microfauna correlated with the Jaramillo subchron^14^. Kahlke^15^ correlated the site of Untermassfeld (Germany) with the Jaramillo subchron. The normal polarity sequence detected in the archaeological deposits from Le Vallonnet (France) was first correlated with the Jaramillo subchron^16^. However, later U-Pb data, in combination with paleomagnetic constraints, associated it with the somewhat older Cobb Mountain subchron^5^.

In Spain, the Jaramillo subchron has been directly identified at the section of Torrent de Vallparadís (Terrassa, northeastern Spain) with the fossiliferous levels EVT10 and EVT12^8^. So far, it was the only section in the Iberian Peninsula where this normal event has been reliably identified. Despite the excellent continuous biostratigraphic succession of the interval around the Jaramillo subchron recorded in both Atapuerca sites of Sima del Elefante (TE) and Gran Dolina (Burgos, Spain), coeval fossiliferous levels with this geomagnetic subchron have not been directly identified^9,17,18^. Similarly, although the Guadix-Baza Basin (Granada, southern Spain) presents one of the best continental records from the late Miocene to the middle Pleistocene in western Europe, a biostratigraphic gap is present covering the coeval Jaramillo deposits^19,20^.

**Figure 1:**
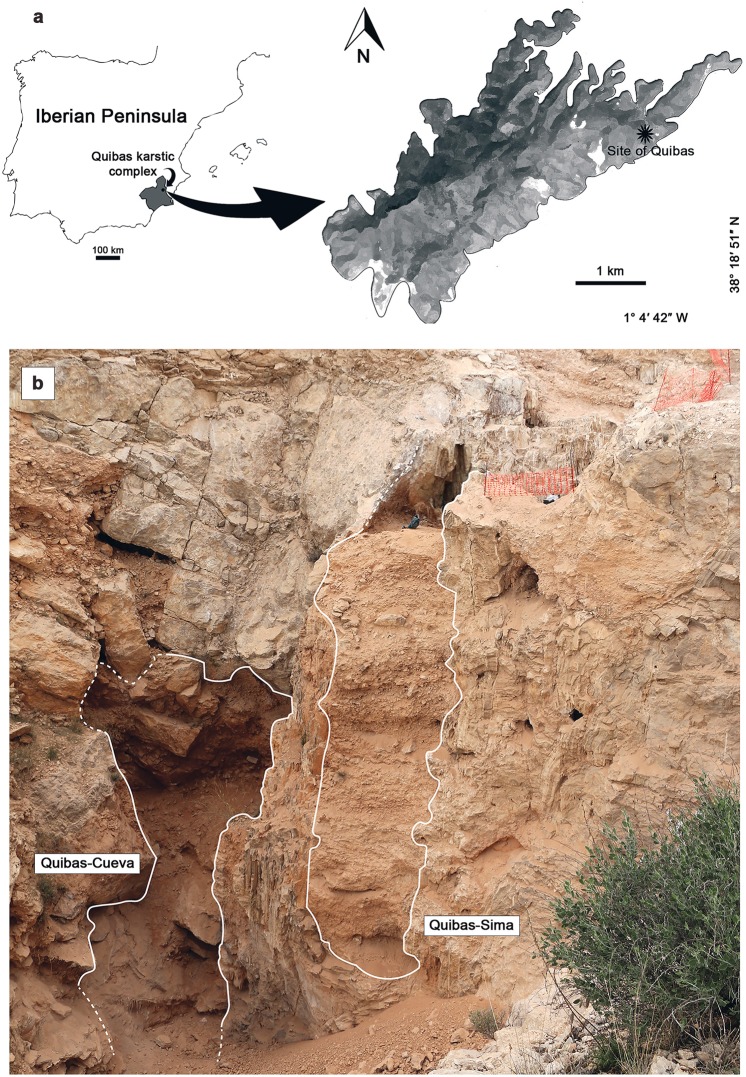
Geographic and structural context of the Quibas site. (**a**) Geographic location of the Quibas karstic complex. (**b)** View of the two main structures of the Quibas site in 2015.

The site of Quibas (Murcia, Spain) (Fig. 1a) is a karstic complex of cavities filled by sediments of early Pleistocene age (see “The site of Quibas” in Supplementary Information for details). The main structures of this complex with paleontological record consist of a gallery called Quibas-Cueva (QC; up to 5 m wide, 9 m high and more than 30 m in length) and a vertical shaft known as Quibas-Sima (QS; 12 m deep and up to 2 m wide) (Fig. 1b). The Quibas-Sima sequence contains seven distinct detritic units: QS-1 to QS-7 (see “Lithostratigraphic units” in Supplementary Information, and Supplementary Figs. S1 and S2). The lowest unit has been subdivided from the base to the top into QS-1.1, QS-1.2 and QS-1.3. The upper units, QS-5 and QS-6 yielded no fossils other than gastropods, while all the underlying units and the uppermost one contained as well vertebrate remains.

Paleomagnetism and in particular magnetic reversal stratigraphy, coupled with biochronology, provides very often a solid time framework in sedimentary rocks. The aim of the present article is to provide an overview of the microvertebrate succession recovered from the Quibas-Sima sequence, as well as to date accurately this section based on its magnetostratigraphic and paleontological data, the later also including the Quibas-Cueva fragmentary record.

## Magnetostratigraphy

**Figure 2:**
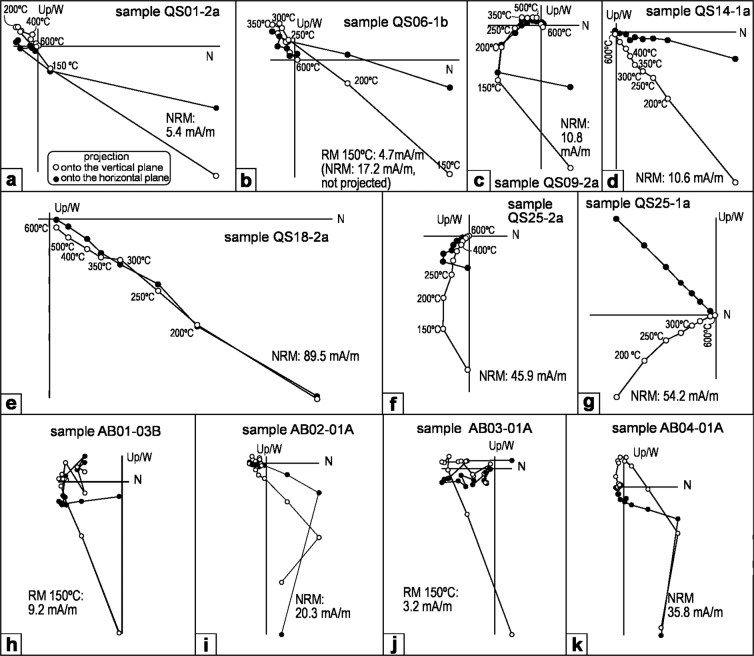
Orthogonal demagnetization plots from the Quibas-Sima section (**a**–**g**, see location in Fig. 3) and Quibas-Cueva (**h**–**k**, see location in Fig. S3). (**a**–**c)** Indicate reverse polarities, and (**d,e)** indicate normal ones. (**f**,**g)** Samples are of undefined polarity. (**h–k)** samples (Quibas-Cueva) are of reverse polarity. Abbreviations: NRM, Natural Remanent Magnetization (room temperature); RM, Remanent Magnetization at a specific temperature.

**Figure 3:**
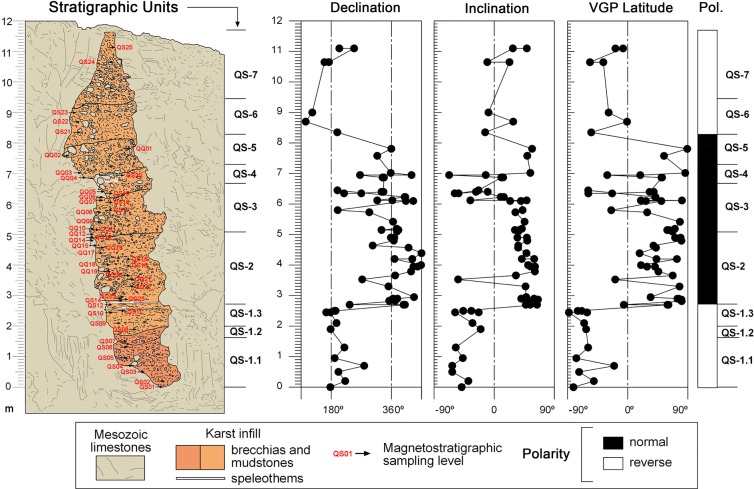
Stratigraphic units (coincident with micromammals sampling units) and magnetostratigraphic results from the Quibas-Sima section.

The general paleomagnetic results from the Quibas-Sima permit a magnetostratigraphic correlation. Samples always display enough intensity to be measured, although in the QS-1 subunits it is generally lower (between 5 and 10 mA/m) compared to the higher values measured in the rest of the succession (reaching values generally between 10 and less than 100 mA/m). During demagnetization, no major shifts in susceptibility were detected and the resulting orthogonal plots display a stable behavior with up to three components. In some samples, an initial viscous and secondary component is observed from room temperature to around 150 °C which is likely to be an overprint of the present-day/recent geomagnetic field. At temperatures between 150 °C and 250–300 °C, a low temperature component is detected (Fig. 2), while a high temperature one can be detected between 250–300 °C and 600 °C. This high temperature component is always directed towards the origin (demagnetization state) and is considered the characteristic one to derive the inclination and declination values of the paleofield at the moment of rock formation. The substantial amount of magnetization above 600 °C observed in the demagnetization plots, and Isothermal Remanent Magnetization (IRM) acquisition curves, reveal the presence of hematite as carrier of magnetization in the high temperature component. The demagnetization plots from the Quibas-Sima section display both reverse polarities (such as Fig. 2a–c), and normal ones (such as Fig. 2d,e). The few samples from Quibas-Cueva (see “Paleomagnetism” in Supplementary Information, and Fig. S3) show only reverse polarities (Fig. 2h–k). In some specimens, unconsistent values are observed, such as having positive inclination and southwards declination (Fig. 2f,g), which may be the result of postdepositional processes such as tilting or reworking. The values of the characteristic component (declination and inclination) were used to caculate the VGP (Virtual Geomagnetic Poles) latitude. The values of these latitudes are used to build a local magnetostratigraphy. Since each stratigraphic sampling level is typically represented by one or two samples (34 and 16 cases, respectively), no mean per site can be calculated for all the section. Thus, quality assessment of samples is carried out by integrating all data belonging to each stratigraphic/paleontologic unit (see Figs. 3 and S4).

In units QS-1.1, QS-1.2 and QS-1.3, the observed polarity is mainly reverse, with most of the samples grouped around consistent values (i.e., south-directed and negative inclinations). On the other hand, in units QS-2 and QS-3 clear normal polarities are detected, with consistent mean values poiting northwards and having positive inclinations. QS-4 is less consistent, but VGP latitudes are dominantly normal (Fig. 3). QS-5 has consistent normal polarities and VGP latitudes. In units QS-6 and QS-7 few samples could be obtained and, although results may be rather dispersed, VGP latitudes are reverse. Regarding the few samples taken at the base of the Quibas-Cueva structure, they showed clear reverse polarities.

## Small Vertebrate Succession

**Figure 4:**
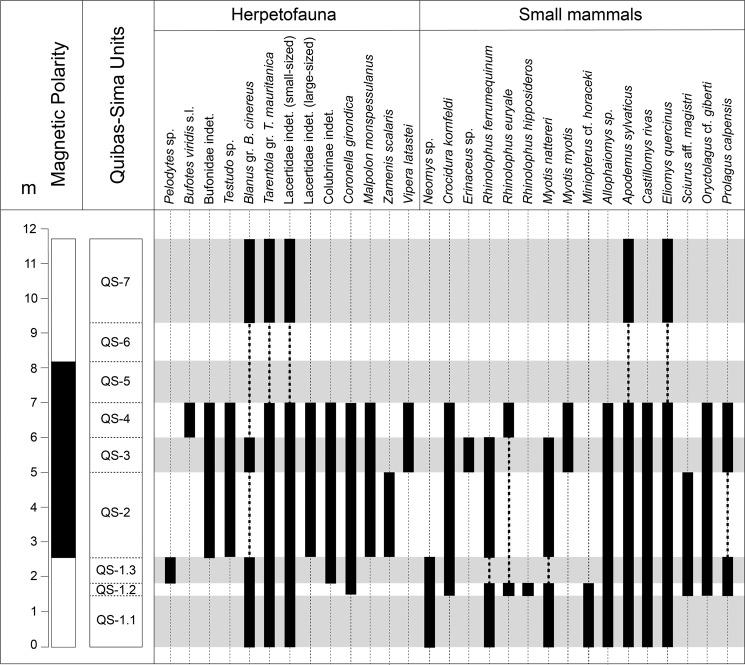
Distribution chart of the taxa found in the Quibas-Sima section.

**Figure 5:**
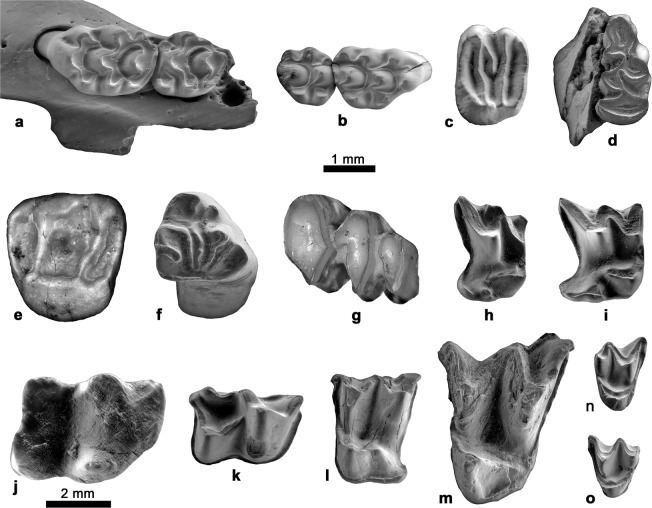
ESEM photographs of representative small-mammal teeth from Quibas-Sima. (**a**) Left maxilla of *Apodemus sylvaticus* with M1 and M2 from QS-3, IPHES-QS-3-R/P25. (**b)** Right M1 and M2 of *Castillomys rivas* from QS-1.3, IPHES-QS-1Z-R/J1. (**c)** Left M1-2 of *Eliomys quercinus* from QS-1.2, IPHES-QS-1A-R/G6. (**d)** Left M3 of *Allophaiomys* sp. from QS-1.1, IPHES-QS-1Z-R-K32. (**e)** Left P4 of *Sciurus* aff. *magistri* from QS-1.2, IPHES-QS-1A-R/AB1. (**f)** Left upper molariform of *Oryctolagus* cf. *giberti* from QS-3, IPHES-QS-3-L/V8. (**g)** Right p3 and p4 of *Prolagus calpensis* from QS-3, IPHES-QS-3-L/V12. (**h)** Right M1 of *Crocidura kornfeldi* from QS-1.3, IPHES-QS-1Z-I/AC2. (**i)** Right M1 of *Neomys* sp. from QS-1.3, IPHES-QS-1Z-I/AC1. (**j)** Right m2 of *Erinaceus* sp. from QS-3, IPHES-QS-3-I/AC1. (**k)** Right m1 of *Rhinolophus ferrumequinum* from QS-3, IPHES-QS-3-Q/V15. (**l)** Left M1 of *Miniopterus* cf. *horaceki* from QS-1.2, IPHES-QS-1A-Q/W2. (**m)** Left M2 of *Myotis myotis* from QS-4, IPHES-QS-4-Q/Z1. (**n**) Right M3 of *Rhinolophus euryale* from QS-1.2, IPHES-QS-1A-Q/W18. (**o**) Left M3 of *Rhinolophus hipposideros* from QS-1.2, IPHES-QS-1A-Q/W19. The 2 mm scale bar goes with figure (**j**) and the 1 mm scale bar goes with the remaining figures.

The small vertebrate assemblage from the Quibas-Sima section comprises amphibians (Bufonidae, Pelodytidae), reptiles (Testudinidae, Gekkonidae, Blanidae, Lacertidae, Colubridae, Viperidae) and, among mammals, insectivores (Soricidae, Erinaceidae), bats (Rhinolophidae, Vespertilionidae), rodents (Arvicolinae, Muridae, Gliridae, Sciuridae) and lagomorphs (Leporidae, Ochotonidae) (Figs. 4 and 5).

The lower unit from the Quibas-Sima sequence (QS-1.1, QS-1.2, QS-1.3), is in general characterized by an association which presents a diversified small vertebrate assemblage, including anurans (*Pelodytes* sp.), lizards (*Blanus* gr. *B. cinereus, Tarentola* gr. *T. mauritanica*, a small sized Lacertidae), snakes (*Coronella girondica*), insectivores (*Neomys* sp., *Crocidura kornfeldi*), bats (*Rhinolophus ferrumequinum, Rhinolophus euryale, Rhinolophus hipposideros, Myotis nattereri, Miniopterus* cf. *horaceki*), rodents (*Allophaiomys* sp., *Apodemus sylvaticus, Castillomys rivas, Eliomys quercinus, Sciurus* aff. *magistri*) and lagomorphs (*Prolagus calpensis, Oryctolagus* cf. *giberti*). Many of these elements are related to living forms which indicate a Mediterranean climate, such as *Blanus cinereus, Tarentola mauritanica, Rhinolophus ferrumequinum* or *Rhinolophus hipposideros*.

Among the insectivores, the presence of the shrew *Neomys* in the Iberian small mammal assemblages becomes rather frequent close to the transition from the early to middle Pleistocene, as it is the case of the latest early Pleistocene sites of Almenara-Casablanca 3 and Gran Dolina TD3-TD8^21,22^, replacing the neomyini species *Asoriculus gibberodon*, present in many early Pleistocene sites such as Fuente Nueva 3 and Barranco León 5 (Guadix-Baza Basin) and Sima del Elefante (TE7-TE14), which record the earliest hominin presence in Western Europe^9,20,23–25^. All the extant *Neomys* species have aquatic habits. This is why the occurrence of the genus is usually considered as indicative of the presence of water bodies in the surrounding area. Up to date, the occurrence of *Neomys* in Quibas-Sima is probably the oldest record of this genus in Spain. Other than the presence of a *Neomys* species in the subunits QS-1.1, QS-1.2 and QS-1.3, the insectivore assemblage is clearly dominated by the species *Crocidura kornfeldi*. It is widely known that crocidurine shrews are better adapted to milder and more arid climates than their soricine counterparts. Since its entry in the Iberian Peninsula at about 1.5 Ma, *Crocidura kornfeldi* persisted until the early-middle Pleistocene transition in sites such as Cueva Victoria^26^, Almenara-Casablanca 3^21^ and Gran Dolina TD3-TD8^9^.

In the case of extinct rodent species, development of ever-growing molars in the microtine *Allophaiomys* sp. lineages or the development of longitudinal ridges between cusps (stephanodonty) in the murine *Castillomys rivas*, have been interpreted as dental adaptations to foraging in open grasslands^27–29^. However, the presence of a forested habitat is attested by the occurrence of *Rhinolophus hipposideros*, *Apodemus sylvaticus* and, most specially, the squirrels of the species *Sciurus* aff. *magistri*. Moreover, the dormouse *Eliomys quercinus* is particularly frequent in these lower levels of Quibas-Sima, reaching a 14% at the subunit QS-1.3. This species currently occupies habitats of low-density deciduous and conifer forests, rocky areas with some vegetation and woodland edges from the Iberian Peninsula to the Ural Mountains. Areas with abundant herbaceous vegetation seem to be a limiting factor on its presence^30^. The presence of favourable environmental conditions are supported by the number of bat species identified in this level (up to five species). The occurrence of *Blanus* gr. *B. cinereus* in QS-1.1, QS-1.2 and QS-1.3, a species linked to humid and covered areas, together with *Pelodytes* sp. in QS-1.3 would probably suggest locally wetter and more forested environmental conditions than in the overlying units in the sequence. This is consistent with the record of the anguid *Ophisaurus manchenioi* in the base of Quibas-Cueva, correlated with QS-1, a taxon linked to subtropical humid conditions^31^.

The unit QS-2 retains most of the species found in QS-1. However, the parsley frog *Pelodytes* sp., the shrew *Neomys* sp. (the insectivores becoming represented only by *Crocidura kornfeldi*) and the bats *Rhinolophus hipposideros* and *Miniopterus* cf. *horaceki* do not reappear at any of the remaining units of Quibas-Sima. Other taxa absent in QS-2 such as the bat *Rhinolophus euryale* or the lagomorph *Prolagus calpensis* reappear in more recent units of the Quibas-Sima sequence. Among the reptiles, the punctual presence of the snake *Zamenis scalaris*, and the first occurrence of chelonids (*Testudo* sp.), a large-sized lizard and the snake *Malpolon monspessulanus* must be pointed out, besides *Tarentola* gr. *T. mauritanica*, *Coronella girondica* and a small-sized lizard. The representatives of the genus *Testudo* are terrestrial chelonians that prefer semi-arid habitats, with Mediterranean vegetation and open areas with a strong insolation such as the Mediterranean forest and shrub. *Malpolon monspessulanus* is a thermophilous snake very well adapted to all types of Mediterranean ecosystems, showing a certain preference for shrubland of warm and dry climate^32,33^.

Although the presence of *Apodemus sylvaticus*, *Sciurus* aff. *magistri* or *Zamenis scalaris* points to the persistence of forest or open-forest formations at this unit, an environmental change between QS-1 and QS-2 is attested by the extirpation of *Neomys* sp. and the occurrence of species with preferential affinity for more open habitats such as *Malpolon monspessulanus* and *Testudo* sp.

A number of changes in the small vertebrate association is observed in QS-3 and QS-4 when compared to the association at QS-2. The snake *Vipera latastei* and the anuran *Bufotes viridis* appear for the first time. *Vipera latastei* is a discontinuously distributed Iberian and North African species that preferentially lives in deteriorated scrubs and mountainous dry areas, showing special affinity for open areas with little or no vegetation such as scree environments^34^. Green toads (*Bufotes viridis* sensu lato) belong today to a widespread group of closely related species with a range that extends from eastern France and Italy to central Asia, including northern Africa and numerous Mediterranean islands. They inhabit mesic to arid environments, from subtropical to cold temperate, ranging in altitudes from below sea level in Israel to more than 4000 m.a.s.l. in the Himalayas^35^. Fossils belonging to this group have been reported in late early Pleistocene sites in Spain, such as Barranco León^36,37^, Almenara-Casablanca 3^38^ and Cueva Victoria^39^. It is also known in northern Africa at the early Pleistocene site of Ahl al Oughlam (Casablanca, Morocco^40^). The last record of *Bufotes viridis* so far comes from the late early Pleistocene of Cueva Victoria, being absent since then from the continental record of the Iberian Peninsula.

Among the insectivores, unit QS-3 records the presence of *Erinaceus* sp. and *Crocidura kornfeldi*. *Erinaceus* is usually considered as a generalistic taxon, able to occupy habitats with different environmental conditions, although it usually avoids extreme conditions such as in deserts or ever-frozen regions. Concerning bats, QS-3 and QS-4 record the entry of *Myotis myotis* and the reappearance of *Rhinolophus euryale*. Perhaps the most significant change compared to the lower units of Quibas-Sima is the absence of the squirrels of the species *Sciurus* aff. *magistri*, which is probably indicative of a decrease in arboreal cover. This observation is consistent with the presence of species preferentially linked to open-land environments, such as *Testudo* sp., *Vipera latastei*, *Malpolon monspessulanus* or *Erinaceus* sp.

Vertebrates are apparently absent from the upper units of Quibas-Sima (QS-5, QS-6) with the exception of the uppermost part of the sequence (QS-7), which delivered some scarce remains of *Blanus* gr. *B. cinereus, Tarentola* gr. *T. mauritanica*, a small-sized lacertid, *Apodemus sylvaticus* and *Eliomys quercinus*.

Therefore, the faunal succession of Quibas-Sima points to a progressive reduction of tree cover from QS-1 to QS-4. This section is clearly divided into two paleoenvironmental episodes: a first one (unit QS-1) is characterized by the presence of a forested mosaic habitat with moderately moist conditions, and a second one (QS-2, QS-3 and QS-4) is indicative of a more open landscape with relatively drier environmental conditions.

## Magnetobiostratigrafic Correlation

The joint presence of *Castillomys rivas* and an archaic representative of *Allophaiomys* in Quibas-Sima and the lower part of Quibas-Cueva places them in the late early Pleistocene (Biharian mammal age)^41–43^. The age of these two sections can be finely constrained on the basis of the correlation of the obtained polarities with the Geomagnetic Polarity Time Scale and the available biostratigraphic data from other Spanish late early Pleistocene sites such as Fuente Nueva-3 (FN-3, Guadix-Baza Basin), Vallparadís (Terrasa, NE Spain), Cueva Victoria (SE Spain) and Gran Dolina (TD, Atapuerca karstic complex). The entire lower part of Quibas-Cueva records a reversed polarity interval, whereas the Quibas-Sima section records a reversed-normal polarity sequence. So while the unit QS-1 is included in a reversed interval, the units QS-2 to QS-5 have provided a normal polarity. To this is added the record of a second reverse polarity in QS-6 and QS-7.

According to the micromammal content, the reversed Quibas-Cueva section can be correlated with the reversed base of Quibas-Sima, both of which share the presence of *Neomys* sp. and *Castillomys rivas*^44,45^, as well as *Apodemus sylvaticus*, *Sciurus* aff. *magistri*, *Allophaiomys* sp. and *Eliomys quercinus*, among others. Paleomagnetic data from FN-3 show that this site is placed in a reversed chron identified as upper Matuyama^46^. The radiometric data indicated that FN-3 is placed below the Jaramillo subchron^7^. Duval *et al*.^7^ and Lozano-Fernández *et al*.^47^ dated FN-3 to ∼1.2 Ma. Quibas-Sima shares with this site the presence of an endemic species of *Allophaiomys* (*Allophaiomys* sp.), which suggests a roughly similar age. However, as we have mentioned previously, the occurrence of *Neomys* in Quibas-Sima probably represents the oldest record of this genus in Spain. This genus is still absent in FN-3 where *Asoriculus* has not yet been replaced by *Neomys*, which indicates that QS-1 is somewhat younger. In this way, the correlation of the reversed polarity observed in the lower part of Quibas-Sima and Quibas-Cueva to the pre-Olduvai Matuyama interval is discarded. Otherwise, the last occurrence of *Castillomys rivas* is recorded in the level Cúllar-Baza B^19^. It is located in the lower part of the section of Cúllar-Baza, Guadix-Baza Basin. Magnetobiostratigraphic correlations from Cúllar-Baza placed the reversed interval of this part of the section in the uppermost Matuyama chron, immediately before the Matuyama-Bruhnes boundary (between 0.99 and 0.78 Ma)^19^. *Castillomys* is already absent in the lowermost levels of Gran Dolina (TD4 to TD7, shortly after the Jaramillo subchron but before the Matuyama-Bruhnes boundary at 0.9 Ma^9,18,48^). Thus, this genus became extinct before the early-middle Pleistocene boundary. The presence of *Castillomys rivas* in Quibas-Sima allows us to discard the assignament of the upper normal polarity from QS-2 to QS-5 to the Bruhnes chron. This is confirmed by the record of a reverse polarity in the overlying QS-6 and QS-7. In addition, the arvicolid species found in the studied section is already absent from the nearby site of Cueva Victoria where it has been replaced by the more advanced arvicolid *Allophaiomys chalinei*. Moreover, the cricetid *Allocricetus bursae* is still absent in Quibas-Sima and present in Cueva Victoria. The latter site has been placed in the upper Matuyama chron between Jaramillo and Brunhes and correlated with MIS-22, at about 0.9 Ma^49^.

The paleontological sites of Quibas are also older than EVT12 and EVT10, from the section of Vallparadís. These two levels are placed in a normal polarity interval which has been correlated with the Jaramillo subchron^8^. In these levels *Iberomys huescarensis* is already present, a microtine species which is common in latest early Pleistocene localities such as those of Gran Dolina (TD3/4, TD5, TD6 and TD8), in Atapuerca, or Huéscar and Loma Quemada, in the Guadix-Baza Basin. However, this species has not been recorded at any level of the entire sequence of Quibas-Sima, pointing to an older age. Therefore, contrary to the Vallparadís section, the Quibas-Sima sequence covers the lower boundary between the upper Matuyama and Jaramillo chrons. The entry of *Iberomys huescarensis* must have taken place within the Jaramillo subchron, some time between the deposition of QS-4 from the Quibas-Sima sequence and EVT12 from the Vallparadís sequence.

**Figure 6:**
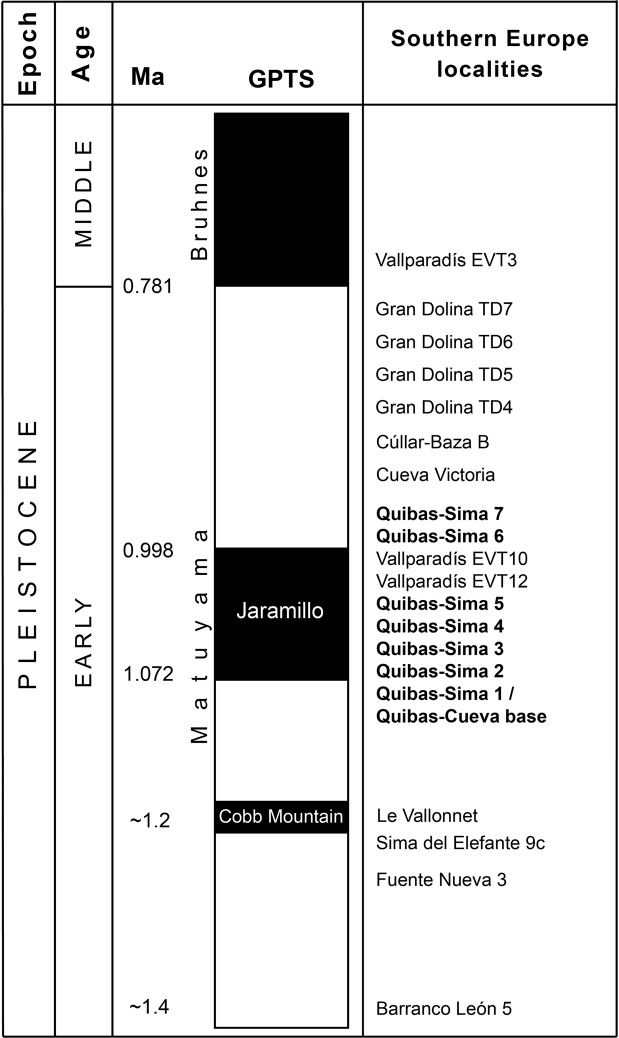
Magnetobiostratigraphic correlation of several European localities^2,7,8,17–19,46,49^ and location of the Quibas-Sima units (QS-1 to QS-7) and the base of Quibas-Cueva. GPTS (Geomagnetic Polarity Time Scale) shows Bruhnes, and two normal polarity intervals within Matuyama: subchrons Jaramillo (1.07–0.99 Ma) and Cobb Mountain (1.22–1.19 Ma)^53^.

Therefore, the age of the base of Quibas-Cueva and Quibas-Sima (QS-1) can be roughly constrained between 1.2 (age of FN-3) and 1.07 Ma, whereas the normal polarity detected at QS-2, QS-3, QS-4 and QS-5 can be correlated with the subchron Jaramillo, between 1.07 and 0.99 Ma (Fig. 6). This correlation is consistent with the macromammal association from Quibas-Cueva described by Montoya *et al*.^44,45^, Piñero and Alberdi^50^ and Alba *et al*.^51^, which includes two species of horses *(Equus altidens* and *Equus suessenbornensis*) and the macaque *Macaca sylvanus florentina*.

## Conclusions

The Quibas-Sima section records a polarity change from a reverse interval (QS-1) to a normal one (QS-2, QS-3, QS-4, QS-5) plus a second reverse one (QS-6 and QS-7). This normal polarity interval bounded by reverse polarities, is identified as the Jaramillo subchron. In Europe, vertebrate assemblages unambiguously assigned to Jaramillo subchron are very scarce. Up to now, the Quibas-Sima sequence represents the longest and most complete pre-Jaramillo and Jaramillo continental vertebrate succession from this continent. This section thus provides a unique chance to increase our knowledge on the paleoenvironmental and faunal context of the time interval comprised between 1.2 and 0.99 Ma. This time span is the prelude to the transition from the early to the middle Pleistocene, marked by major changes in the climatic cyclicity of the Earth. Under these circumstances, the information provided from the study of the Quibas-Sima sequence is an important contribution to our understanding of the climatic and faunal events that occurred at the beginning of the early-middle Pleistocene transition. A significant change in the small vertebrate composition has been observed from QS-1 to QS-4. Among the small mammals, this change involved the extirpation of species associated to woodland habitats and water bodies such as *Sciurus* aff. *magistri* and *Neomys* sp., respectively, as well as a drop in the bat diversity. Among reptiles, it involved a shift towards species with a preference for dry and open areas, such as *Testudo* sp., *Vipera latastei* and *Malpolon monspessulanus*. Therefore, it seems that close to the onset of the Jaramillo subchron, a progressive environmental change involving a reduction of tree cover and spreading of shrublands or grasslands took place. At the Quibas-Sima section, this change is coincident with the transition from MIS 31 to MIS 30. While in northern latitudes the transition from interglacial to glacial conditions involved a decrease in global temperatures. It seems that this was not the case for lower latitudes such as the southern part of the Iberian Peninsula. While interglacial conditions such as MIS 31 favoured at these latitudes the extension of woodlands or open woodlands, during glacial times as was the case of MIS 30, all evidence points to increased aridity leading to a reduction of forested formations and the extension of open-land habitats.

## Materials and Methods

Most of the small-vertebrate sample was collected from the Quibas-Sima section during the sampling campaing of 2014, but it is also included material recovered during the systematic excavation campaigns of 2015, 2016, 2017 and 2018. All the sediment was water-screening using superimposed 4, 1 and 0.5 mm mesh screens. The Quibas-Sima sample includes 2584 identified small-vertebrate remains corresponding to 29 taxa. QS-1.1 has yielded 26 remains representing 11 species, the material from QS-1.2 comprises 423 small-vertebrate fossils of at least 18 species, the sample of QS-1.3 consists of 831 specimens ascribed to 15 taxa, QS-2 has yielded 141 specimens comprising 18 taxa, the material from QS-3 consists of 796 identified remains representing 21 small-vertebrate species, the sample from QS-4 contains 243 remains assigned to 19 taxa, and QS-7 is the poorest unit with only nine specimens belonging to five taxa. The recovered fossils are currently kept at the Institut de Paleoecologia Humana i Evolució Social (IPHES; Tarragona, Spain). Small-mammal teeth are illustrated by means of micrographs taken with Environmental Scanning Electron Microscopy (ESEM) at the Servei de Recursos Científics i Tècnics de la Universitat Rovira i Virgili (Tarragona).

For this paleomagnetic study at the Quibas karstic complex, the Quibas-Sima section was sampled in some 50 levels with an electrical drill. Each sample was a single core that, if long enough, was cut into several samples. These specimens underwent a paleomagnetic study, accounting for a whole of some 70 studied samples. These samples underwent a stepwise thermal demagnetization and measuring of remanence in a triaxial cryogenic magnetometer at in Laboratory of Paleomagnetism at Universitat de Barcelona (SCT-CSIC). Thermal demagnetiser TSD-1 (Schonstedt) was used for this study, while bulk susceptibility was measured with the KLY-2 susceptibilimeter (Geofyzika Brno). Paleomagnetic components were derived with the Koymans *et al*.^52^ software. A thermal demagnetization protocol including from 8 to 13 steps was applied to the studied samples. This protocol ranged from NRM (Natural Remanent Magnetization) to 600 °C. The basal part of the lateral (cave) section was also sampled with few additional cores.

## Supplementary information

Supplementary Information.
