# Supplementary material for: First continuous pre-Jaramillo to Jaramillo terrestrial vertebrate succession from Europe
Source: Sci Rep. 2020 Feb 5;10:1901. doi: 10.1038/s41598-020-58404-w (PMC7002404; doi:10.1038/s41598-020-58404-w)
Supplement: Supplementary file 1 — Supplementary Information. [file 41598_2020_58404_MOESM1_ESM.docx]

Supplementary Information for “First continuous pre-Jaramillo to Jaramillo terrestrial vertebrate succession from Europe”

Pedro Piñero, Jordi Agustí, Oriol Oms, Hugues-Alexandre Blain, Marc Furió, César Laplana, Paloma Sevilla, Antonio Rosas, Josep Vallverdú

The site of Quibas

The site of Quibas (Abanilla, Murcia) is a karstic complex of cavities filled by sediments of early Pleistocene age. It is located in the vicinity of the town of Cañada de la Leña, on the SE slope of the Sierra de Quibas, in an abandoned limestone quarry (Fig. 1a). The location coordinates are 38° 18′ 51″ N, 1° 4′ 42″ W. The paleontological site has yielded, since its discovery in 1994, fossil remains of more than 70 species of both vertebrates and invertebrates from the late early Pleistocene. The significance of this site lies in its chronology, the great diversity of its fauna and the excellent preservation of the remains. The main structures of this karstic complex with paleontological record consist of a gallery called Quibas-Cueva (QC; up to 5 m wide, 9 m high and more than 30 m in length) and a vertical shaft known as Quibas-Sima (QS; 12 m deep and up to 2 m wide) (Fig. 1b). These two cavities are separated by 3-m-thick calcitic speleothems but are connected internally (Fig. S1). Besides these two major infillings, a smaller detritic cavity known as Gruta-1 with small vertebrate remains has also been described^41,54^.

The first period of excavations at the Quibas complex took place between 1999 and 2009 and was mainly focused on the Quibas-Cueva structure. The information collected during those years gave place to a number of publications. Preliminary faunal lists were published by Montoya *et al*.^44,45^, including both the vertebrate and invertebrate fossil record. Regarding the macrofauna, several specific taxonomic works have been done^50,51,55–57^. As for the microfauna, Montoya *et al*.^44,45^ included a preliminary identification of the microvertebrate association. Piñero *et al*.^41^ described the rodent assemblage from the cavity known as Gruta1 establishing a biochronological framework, but magnetostratigraphic data were not available. Piñero *et al*.^54^ performed a paleoenvironmental reconstruction based on the rodent assemblage from Gruta1. Blain *et al*.^58^ ascertained the latest presence of agamid lizards from Western Europe. Blain and Bailon^31^ presented a new anguid species (*Ophisaurus manchenioi*), last representative of its genus in Western Europe.

However, so far no studies focused on the Quibas-Sima sequence. Given the indisputable significance of the Quibas site concerning the knowledge of the late early Pleistocene from SW Europe, digging activities in the site were resumed in 2014 with the purpose of characterising the sequence at the Quibas-Sima section from a faunistical and climatic perspective. The different units in the sequence were clearly defined, samples for microfauna were collected at each as well as samples for magnetostratigraphic analyses, which were taken along the entire succession. In later excavation campaigns, an important amount of faunal remains was recovered, thus providing a representative collection of fossils to meet the aims layed out in this paper.

Geological setting

The Sierra de Quibas is a 6-km-long and 2.5-km-wide calcareous massif, mainly of Jurassic limestone and dolomites. It runs in a NE-SE direction in the Middle Subbetic region^59^. This complex was affected by great karstic activity during the Plio-Pleistocene, resulting in many karstic structures. In some of these structures mixed detritic sediments and/or chemical precipitates are found, an example of which is found close to the Quibas outcrop and described here^60^. Thus, the Quibas sites consist of several Pleistocene karstic infillings developed in this quarry of Lower Lias (Jurassic) dolomites. Each infilling has its own particular characteristics; the stratigraphy and sedimentological features of the lower part of the Quibas-Cueva structure were described by Fumanal and Blázquez in Montoya *et al*.^44^ and that of the small cavity Gruta1 by Piñero *et al*.^41^.

The Quibas cave walls show large scallop marks suggesting their phreatic origin. The lithostratigraphic units of Quibas cave infilling are based on the outcrop named Quibas-Sima (Quibas shaft). The sediments found infilling the Quibas sites consist mainly of the typical limestone breccias commonly found in the entrance of caves in which endogenous and exogenous materials are mixed, containing fragments of rock from the roof and walls of the cave together with typical “terra rosa” and soil. Limestone fragments, large gravel and boulders show longitudinal grain size sorting increasing from Quibas-Sima outcrop to Quibas-Cueva. These longitudinal sorting of the boulders and gravels point out to forms and sedimentary process of talus slope and cone deposits.

Lithostratigraphic units

The Quibas-Sima sequence consists of seven distinct detritic units (see Figs. S1 and S2 for details): QS-1 to QS-7. The upper units, QS-5 and QS-6 yielded no fossils other than gastropods, while all the underlying units and the uppermost one contained as well vertebrate remains.


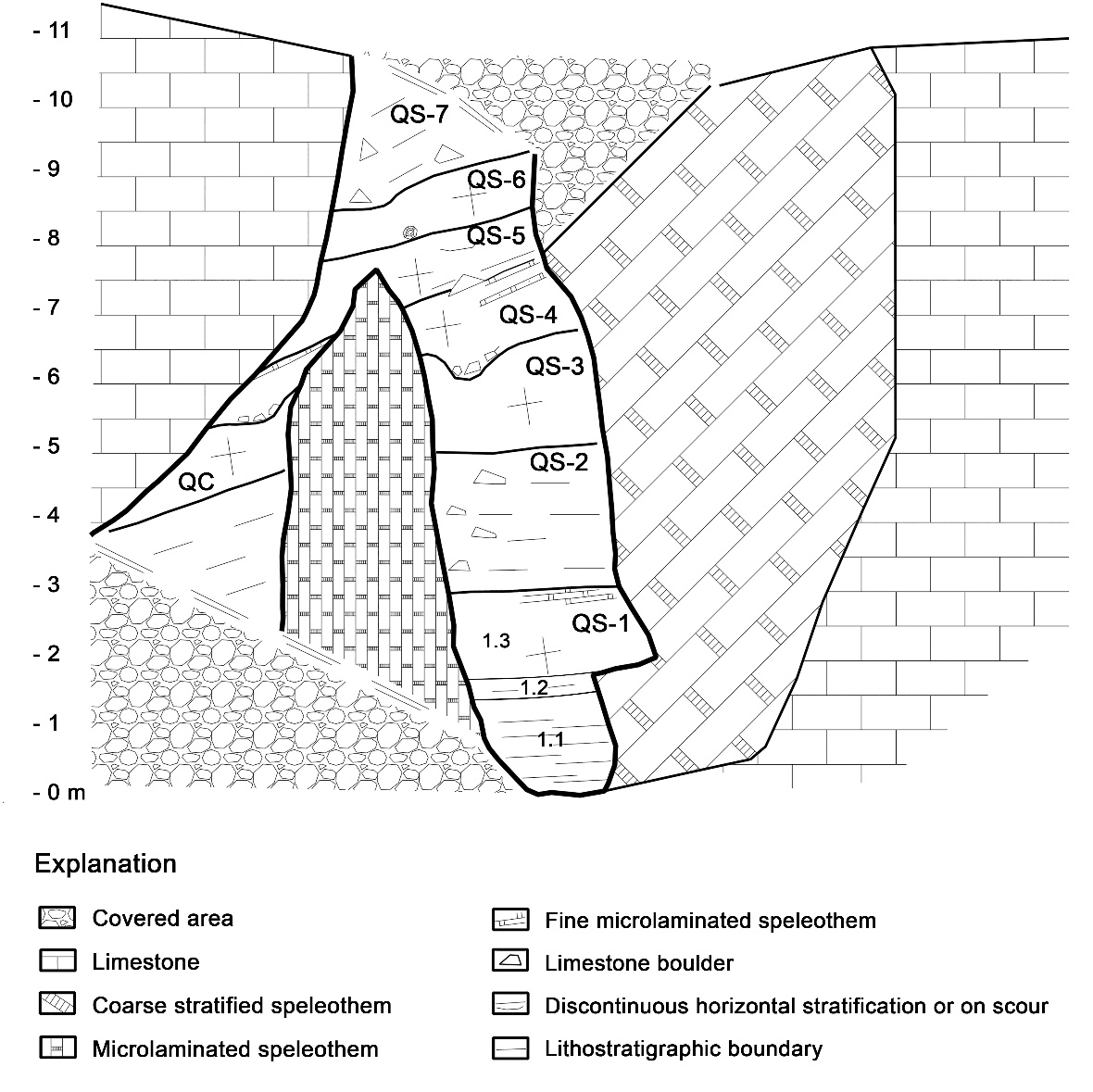


Fig. S1: Profile of the northern sector of the Quibas karstic complex.


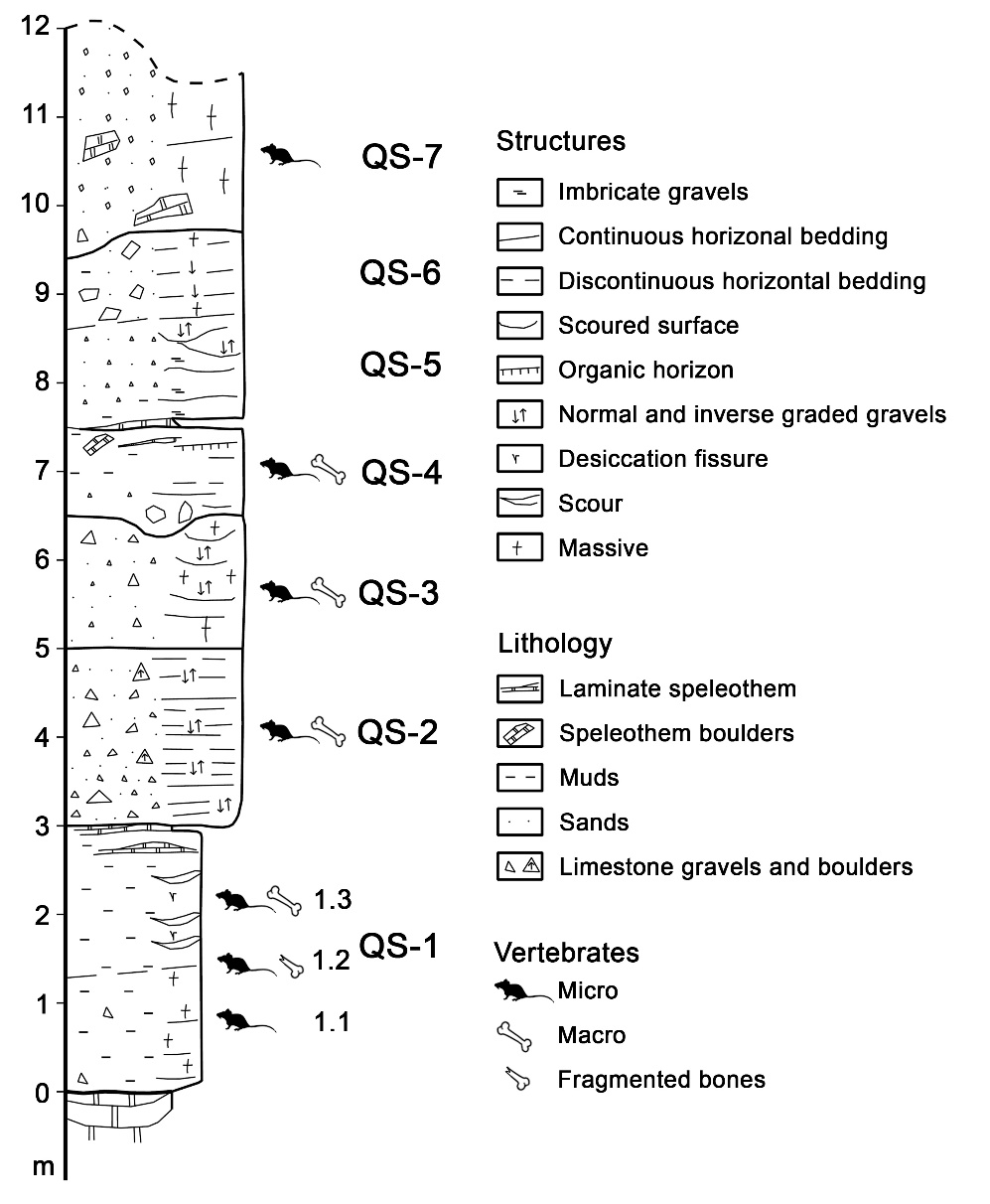


Fig. S2: Lithostratigraphic column of the Quibas-Sima section.

QS-1 lithostratigraphic unit is 3 m thick and contains three subunits of 1.5 m (QS-1.1), 0.3 m (QS-1.2) and 1.2 m (QS-1.3) thick. The sediments of QS-1.1 are made from massive muddy sand of light brown (7.5YR 6/4) color and poorly microstratified. The sediments contain granules, fine gravels of speleothems and small-vertebrate remains. QS-1.2 is formed by reddish detritic sediment rich in clays. It has yielded fragmented bones of large mammals. Microvertebrate fossils are abundant. QS-1.3 has the same color than QS-1.1 and muddy sand texture, and shows microstratification. It is rich in microfauna remains, and has delivered some large mammal bones. There are bioturbations, scours and dissection structures. The upper surface boundary of QS-1 contains a continuous speleothem deposit.

QS-2 is 2 m thick and made from a microstratified microbreccia of light brown muddy sand. The fine and horizontal strata are made from fine and unimodal clast-supported limestone gravel structure. The strata of fine gravel form normal and inverse gradation and contain outsize boulders in vertical position. The microvertebrate fossils are abundant in this unit. It also contains macrovertebrate bones.

QS-3 is a bimodal breccia deposit of 1.5 m thick and contains boulder and gravel clast structures with reddish yellow (5YR 6/6) muddy sand. The boulders are 25 cm size and the gravels are fine (4-6 cm). The structure of the bedsets of breccia is open work. This unit is rich in large and small vertebrates.

QS-4 is a breccia deposit of 1 m thick made from unimodal fine gravel (4-6 cm) and cemented muddy sand of reddish yellow color (7.5YR 6/6). The lower surface boundary contains a scoured surface infilled with boulders. The breccia is microstratified with discontinuous fine strata showing normal and inverse gradation. The lower 0.5 m of the breccia is supported by muddy sand. The upper bedset is made from fine and cemented beds. The upper surface boundary of QS-4 contains a discontinuous deposit of speleothems and a scour with a boulder deposit of speleothems. This unit has yielded both small and large vertebrate fossils.

QS-5 unit is made from well microstratified breccia of 1 m thick. This deposit contains three horizontal and parallel bedsets and two lenticular bedsets close to the upper surface boundary. The lower horizontal bedsets are made of unimodal fine gravel (4-6 cm) in clast support structure with planar fabric or in pale brown muddy sand matrix support structure. The upper lenticular gravel beds show normal and inverse gradation in open work and partly clast support structure. Vertebrates are absent.

QS-6 deposits are breccias made up in a gradual lower surface boundary of 1 m thick. The breccia is microstratified in four bedsets of discontinuous and horizontal strata that contain boulders of roughly 25 cm. The structure of the breccia deposits consists in muddy sand matrix support of pale brown color with normal-inverse gradation (weakly developed). This unit is sterile of vertebrate fossils.

QS-7 breccia is bimodal and cemented with 2.5 m thick. The breccia is poorly stratified. It is made from medium and fine-sized limestone and speleothemic gravel in clast support structure infilled with calcitic pale brown muddy sand. There are two strata of large boulders made from speleothems. This unit has delivered scarce remains of small vertebrates.

Paleomagnetism

The Quibas-Cueva sampling sites (Fig. S3) were drilled in 2001 in scattered outcrops. The stereoplot of the directions of these specimens is shown in Fig. S4 and four demagnetization plots from each sampling level are shown in Fig. 2h–k.


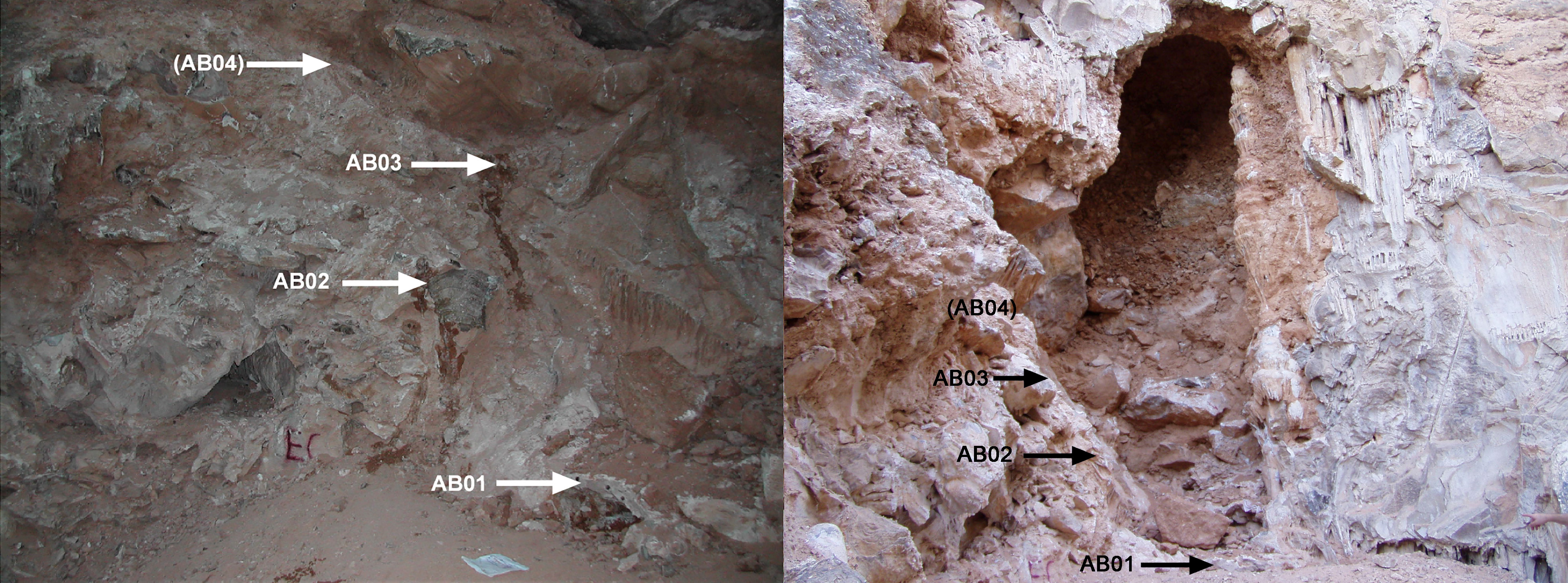


**Fig. S3**: Location of the samples from Quibas-Cueva taken in 2001 (site AB04 is hidden due to the vision).


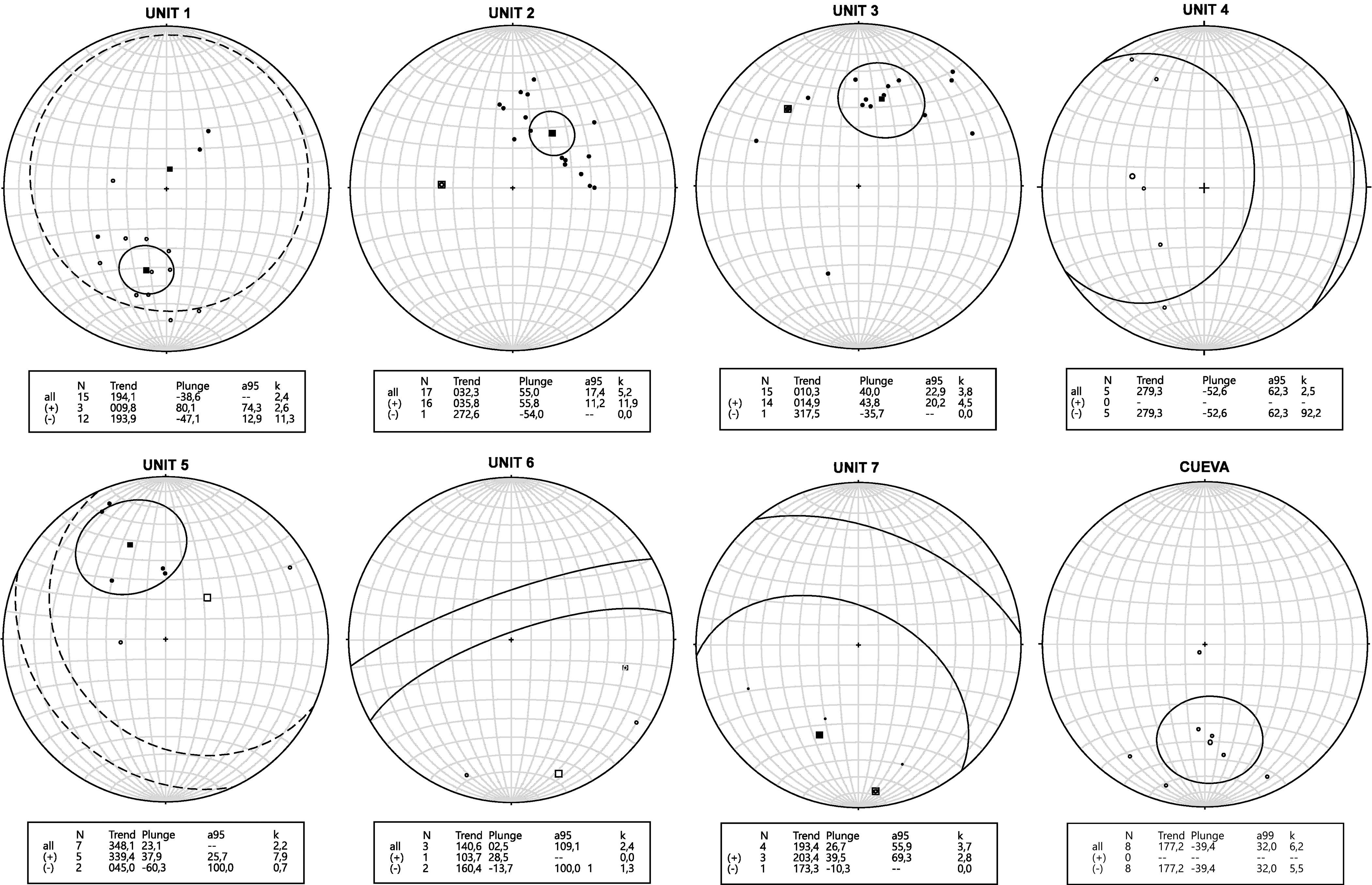


**Fig. S4**: Stereographic projection of paleomagnetic results (specimen declination and inclination) from each stratigraphic unit in Quibas-Sima and base of Quibas-Cueva (bottom to the right). See main text.

References for supplementary information

1. Piñero, P., Agustí, J., Blain, H. -A. & Laplana, C. Paleoenvironmental reconstruction of the Early Pleistocene site of Quibas (SE Spain) using a rodent assemblage. *C. R. Palevol* **15**, 659–668 (2016).
2. Carlos-Calero, J. A., Montoya, P., Mancheño, M. A. & Morales, J. Presencia de *Vulpes praeglacialis* en el yacimiento pleistoceno de la sierra de Quibas (Murcia, España). *Estud. Geol.* **62**, 395–400 (2006).
3. van der Made, J., Carlos Calero, J. A. & Mancheño, M. A. New material of the goat *Capra alba* from the Lower Pleistocene of Quibas and Huéscar (Spain). Notes on sexual dimorphism, stratigraphic distribution and systematic. *Boll. Soc. Paleontol. Ital.* **47**, 13–23 (2007).
4. Pérez-García, A., Murelaga, X., Mancheño, M. A., Rodriguez, A. A. & Romero, G. The tortoises from the Lower Pleistocene palaeontological site of Quibas (Region de Murcia, Spain). *C. R. Palevol* **14**, 589–603 (2015).
5. Blain, H. -A. *et al*. Youngest agamid lizards from Western Europe (Sierra de Quibas, Spain, late Early Pleistocene). *Acta Palaeontol. Pol.* **59**, 873–878 (2014).
6. Rodríguez-Estrella, T., Mancheño, M. A., Romero, G. & Hernández, J. M. Características geológicas de la Sierra de Quibas (Abanilla, Murcia). Su relación con un yacimiento paleontológico pleistoceno. *Geogaceta* **35**, 115–118 (2004).
7. Durán, J. J., López-Martínez, J. & Mancheño, M. Dos registros de espeleotemas pleistocenos de gran potencia en la Península Ibérica: primeros resultados isotópicos. *Bol. Geol. Minero* **115**, 265–270 (2004).
